# Isothermal signal amplification-mediated nanozyme capture on DNA tetrahedra for ultrasensitive amperometric immunoassay of Epstein–Barr virus latent membrane protein 1

**DOI:** 10.3389/fchem.2025.1647535

**Published:** 2025-09-11

**Authors:** Lin Liao, Zhaoxiong Li, Zhanhong Liu, Bin Qiu, Wangyuan Guo

**Affiliations:** 1 Laboratory Medicine Center, The First People’s Hospital of Chenzhou, Chenzhou, Hunan, China; 2 Nasopharyngeal Cancer Diagnosis and Treatment Center, The First People’s Hospital of Chenzhou, Chenzhou, Hunan, China; 3 School of Chemistry, Fuzhou University, Fuzhou, China

**Keywords:** amperometric biosensor, latent membrane protein 1, DNA tetrahedronnanostructure, isothermal signal amplification, nasopharyngeal carcinoma

## Abstract

The overexpression of latent membrane protein 1 (LMP-1), a key oncoprotein encoded by the Epstein-Barr virus (EBV), is closely associated with the development and progression of nasopharyngeal carcinoma (NPC), making it a valuable biomarker for early diagnosis and prognosis. Herein, we report a highly sensitive amperometric immunoassay method for LMP-1 detection based on the strand displacement amplification (SDA)-mediated capture of nanozyme on a DNA tetrahedron (TDN)-modified electrode. In detail, a sandwich immunoassay was carried out on a microplate, followed by sequential capture of streptavidin and biotin-labeled SP-HP, which then initiated SDA in the presence of a nicking enzyme and DNA polymerase. The resulting trigger DNA hybridized with TDN-anchored hairpin probes, exposing terminal digoxin moieties that captured anti-digoxin antibody modified AuPt alloy nanozymes. The nanozymes catalyzed the oxidation of TMB in the presence of H_2_O_2_, generating electroactive oxidized products that were subsequently reduced at the electrode to yield a measurable current signal. The integration of TDNs, SDA, and AuPt nanozymes significantly enhanced sensitivity, achieving a detection limit as low as 47 fg mL^-1^ and a wide linear range (0.1–1,500 pg mL^-1^). The developed method also demonstrated excellent specificity, reproducibility, and applicability in spiked biological samples. This work presents a promising strategy for ultrasensitive and specific detection of EBV-related proteins and expands the utility of nanozyme-based electrochemical immunoassays for clinical diagnostics.

## 1 Introduction

Nasopharyngeal carcinoma (NPC) is a malignant tumor that arises in the epithelial cells of the nasopharynx. It is most prevalent in Southeast Asia, particularly in southern China, where its incidence is significantly higher compared to other regions (Chen et al., 2019; Li et al., 2025). According to recent global cancer statistics, the annual incidence of NPC worldwide is estimated to be around 133,000 cases, with a mortality rate of approximately 75,000 deaths (Bray et al., 2018). Despite its relatively low incidence globally, NPC ranks as one of the leading causes of cancer-related death in endemic areas. Early diagnosis and effective treatment are crucial for improving survival rates, as NPC is often diagnosed at advanced stages when the prognosis is poor (Syafirah et al., 2020). A major etiological factor in the development of NPC is infection with Epstein-Barr virus (EBV), a ubiquitous herpesvirus. The virus has been implicated in the pathogenesis of NPC, with EBV DNA and proteins detected in nearly all NPC tumors (Raab-Traub, 2002).

Among the key viral proteins expressed during EBV infection, latent membrane protein 1 (LMP-1) plays a pivotal role. LMP-1 is a membrane-bound protein that acts as a potent oncoprotein, promoting cell proliferation, survival, and metastasis through multiple signaling pathways (Awasthi et al., 2023). The expression of LMP-1 is strongly associated with the development and progression of NPC, making it a valuable biomarker for both diagnosis and prognosis (Yoshizaki et al., 2013). Recent studies have shown that elevated levels of LMP-1 are linked to poor prognosis in NPC patients, correlating with advanced tumor stages, lymph node metastasis, and lower survival rates (Lao and Le, 2019; Shair et al., 2018). Therefore, detecting and quantifying LMP-1 expression could provide critical information for early diagnosis, risk stratification, and monitoring treatment response in NPC patients (Zhang et al., 2016). However, current methods for detecting LMP-1 expression, such as immunohistochemistry, radioimmunoassay, and immuno-PCR, are often time-consuming, expensive, and require specialized equipment. Developing a novel, reliable, and cost-effective method for detecting LMP-1 would significantly improve the clinical management of NPC, providing a tool for early detection and personalized treatment strategies.

Electrochemical biosensors have emerged as powerful tools for the detection of a wide range of biological targets due to their high sensitivity, rapid response times, and ease of use (Díaz-Fernández et al., 2024; Wu et al., 2023). These sensors work by converting biological interactions into measurable electrochemical signals, making them particularly suitable for applications in point-of-care diagnostics, environmental monitoring, and medical diagnostics (Dong et al., 2024; Wang et al., 2023). Recent advancements have focused on incorporating nucleic acid-based probes to improve the performance of electrochemical biosensors, owing to their high specificity for target molecules (Ye et al., 2024; Zhang et al., 2022). Among these probes, DNA nanostructures, particularly tetrahedral DNA nanostructures (TDNs), have garnered significant attention for their potential to enhance the performance of biosensing systems. TDNs are a type of DNA origami structure with a unique three-dimensional geometry, consisting of four strands that form a stable, tetrahedral shape (Ouyang et al., 2024). TDNs significantly enhance the performance of electrochemical sensors for bio-detection applications. They can improve the uniformity of self-assembled DNA monolayers through enthalpy-entropy compensation, reducing non-specific adsorption and probe entanglement, which increases the efficiency and speed of DNA hybridization (Song et al., 2016). TDNs also enable precise control over interprobe distances, optimizing molecular recognition kinetics and thermodynamics, thus improving sensor sensitivity (Lin et al., 2015). Additionally, TDNs exhibit superior resistance to non-specific protein adsorption, ensuring enhanced specificity and reproducibility. Finally, TDNs are versatile and can be adapted for the detection of various biomolecules, including antibodies and aptamers (Zhang et al., 2023). These features make TDNs an ideal platform for advancing electrochemical biosensing technologies, offering improvements in both sensitivity and specificity for a wide range of bio-detection applications.

Isothermal signal amplification (ISA) techniques are effective methods for amplifying nucleic acids at a constant temperature, eliminating the need for complex thermal cycling (Cao et al., 2023). These techniques are becoming popular alternatives to polymerase chain reaction (PCR) due to their simplicity and suitability for point-of-care testing. Common ISA methods include rolling circle amplification (RCA) (Deng et al., 2023), loop-mediated isothermal amplification (LAMP) (Nam et al., 2023), recombinase polymerase amplification (RPA) (Liang et al., 2024), and strand displacement amplification (SDA) (Feng et al., 2023). The key advantage of ISA methods lies in their ability to achieve high sensitivity and specificity using simple setups that require minimal equipment, making them ideal for on-site diagnostics. Among these techniques, SDA, particularly the version involving the nicking enzyme and DNA polymerase, is especially effective (Carter et al., 2021). In this method, nicking enzyme introduces nicks in double-stranded DNA, while DNA polymerase with strand displacement activity extends from the nick, displacing the downstream DNA strand. This process of nicking and polymerization repeats continuously, resulting in efficient amplification of the target DNA. The SDA method using nicking enzyme and DNA polymerase is highly versatile, as it can be easily combined with other isothermal amplification techniques and can target a broad range of molecules (Niu et al., 2023; Qin et al., 2023). In addition to nucleic acids, it can detect small molecules and proteins, extending its application beyond typical nucleic acid detection. Moreover, this SDA approach supports multiple types of output signals, including fluorescence (Sun et al., 2023), electrochemical (Zhou et al., 2023), electrochemiluminescence (Cui et al., 2023), and colorimetric (Wei et al., 2020) signals, providing flexibility for various diagnostic needs.

Nanozymes are nanomaterials with intrinsic enzyme-like catalytic activity, offering high stability, low cost, and excellent adaptability (Li et al., 2023). Compared to natural enzymes, they exhibit superior robustness, long-term storage stability, and ease of surface modification (Yang et al., 2024; Zhang et al., 2025). In electrochemical biosensing, nanozymes—particularly those mimicking peroxidase or oxidase—are widely used to catalyze redox reactions, enabling sensitive and rapid signal generation or amplification. When combined with recognition elements such as antibodies or aptamers, nanozyme-based electrochemical biosensors provide high selectivity and low detection limits for various biomarkers (Thamilselvan and Kim, 2024). Their high conductivity and large surface area further enhance electron transfer efficiency, making them ideal for constructing miniaturized and portable sensing platforms. These advantages position nanozymes as powerful tools for point-of-care diagnostics and early disease detection. In this study, we developed a highly sensitive amperometric immunoassay for LMP-1 detection, utilizing an SDA-mediated strategy to capture nanozymes on a DNA tetrahedron-modified sensor interface. LMP-1 was recognized through a sandwich immunoassay performed on a microplate, in which a biotinylated secondary antibody, in combination with the biotin–streptavidin (SA) system, enabled the immobilization of a self-priming hairpin probe (SP-HP). In the presence of a nicking endonuclease and DNA polymerase, SP-HP initiated autonomous amplification, generating abundant trigger DNA fragments. These fragments hybridized with hairpin probes anchored on the gold electrode via tetrahedral DNA nanostructures (TDNs), causing their opening and exposing terminal digoxin groups. The digoxin then captured anti-digoxin antibody-conjugated AuPt alloy nanozymes (Ab-AuPt), which possess excellent peroxidase-like activity. Using TMB as the electrochemical substrate, LMP-1 levels were quantified through amperometric measurement. The combination of SDA-driven amplification, nanozyme catalysis, and the TDN-modified electrochemical interface provided enhanced signal transduction and enabled ultrasensitive detection of LMP-1.

## 2 Experimental

### 2.1 Materials and chemicals

Vent^®^ (exo-) DNA Polymerase and Nt. BstNBI were purchased from New England Biolabs (NEB). Enhanced K-Blue TMB substrate was obtained from Neogen Corporation (United States). DNA marker (25–500 bp), deoxynucleotide triphosphates (dNTPs), tris (2-carboxyethyl) phosphine hydrochloride (TCEP), and streptavidin were supplied by Sangon Biotech Co., Ltd. (Shanghai, China). LMP-1 antibody-coated microplates and LMP-1 were acquired from Shanghai Fusheng Industrial Co., Ltd., while detection antibodies were sourced from Abcam. Biotin-labeled goat anti-rabbit IgG (second antibody) were obtained from Beyotime Biotechnology (Shanghai, China). Chloroauric acid and chloroplatinic acid were purchased from Sinopharm Chemical Reagent Co., Ltd. (Shanghai, China). HPLC-purified DNA oligonucleotides were synthesized by Sangon Biotech Co., Ltd. (Shanghai, China), and the corresponding sequences are listed in Table 1. Ultrapure water (18.2 MΩ cm) was prepared using a Milli-Q system.

**TABLE 1:** The sequences of oligonucleotides used in this work.

Name	Sequences (5′to 3′)
Strand A	ACATTCCTAAGTCTGAAACATTACAGCTTGCTACACGAGAAGAGCCGCCATAGTATTTTTTGACCTGTGCAGCGATTATTATTACACAGGTC-digoxin
Strand B	SH-TATCACCAGGCAGTTGACAGTGTAGCAAGCTGTAATAGATGCGAGGGTCCAATAC
Strand C	SH-TCAACTGCCTGGTGATAAAACGACACTACGTGGGAATCTACTATGGCGGCTCTTC
Strand D	SH-TTCAGACTTAGGAATGTGCTTCCCACGTAGTGTCGTTTGTATTGGACCCTCGCAT
Self-primer hairpin probe	GAC​CTG​TGC​AGC​GAC​CTA​GAC​TCG​TCA​GCG​TAG​TCC​GGG​TAC​TGT (-biotin)GCATGCCGGACTACGCTGAC

### 2.2 Synthesis of AuPt alloy nanoparticles and Ab-AuPt nanozymes

A mixture of ultrapure water (50 mL), chloroplatinic acid (0.38 mL, 10 mg mL^-1^), and chloroauric acid (0.3 mL, 10 mg mL^-1^) was prepared in a 100 mL flask and heated under stirring until boiling for 10 min. Subsequently, trisodium citrate (0.2 mL, 100 mg mL^-1^) was introduced, and the reaction proceeded under reflux for 30 min. During this process, the solution gradually changed from nearly colorless to black, indicating the formation of AuPt alloy nanoparticles. Afterward, the resulting AuPt alloy nanoparticle dispersion was naturally cooled to room temperature and stored at 4 °C until use.

To conjugate the anti-digoxin antibody, the pH of the colloidal AuPt solution was adjusted to approximately 8.5 using 0.1 M potassium carbonate (K_2_CO_3_). A defined concentration of antibody (typically 10 μg mL^-1^ in PBS) was slowly added to the AuPt solution under gentle stirring, followed by incubation at room temperature for 60 min to promote effective binding. After conjugation, bovine serum albumin (BSA, final concentration 1%) was added as a blocking agent to passivate the unmodified surfaces of the nanoparticles, and the mixture was incubated for an additional 30 min. The antibody-conjugated AuPt nanoparticles were then collected by centrifugation at 10,000 rpm for 10 min, and the resulting pellet was resuspended in PBS. The final antibody-labeled AuPt conjugates (Ab-AuPt) were stored at 4 °C until use.

### 2.3 Preparation of tetrahedral DNA nanostructures-modified gold electrode (TDNs/AuE)

Before modification, the gold electrode (AuE) underwent sequential polishing on a nylon pad with 0.5 μm and 0.3 μm alpha alumina powders, each step followed by thorough rinsing with double-distilled water. To eliminate potential organic contaminants, the electrode was immersed in a piranha solution (H_2_SO_4_/30% H_2_O_2_, 7:3 v/v) for 15 min and subsequently rinsed thoroughly with double-distilled water. Electrochemical activation of electrode interface was then performed in 0.5 M H_2_SO_4_ using cyclic voltammetry (scanning from −0.3 to 1.55 V) until a stable oxidation peak characteristic of gold was obtained.

Tetrahedral DNA nanostructures (TDNs) were assembled according to a previously reported method. Four single-stranded DNA oligonucleotides (strands A, B, C, and D) were custom-synthesized and HPLC-purified by Sangon Biotech (Shanghai, China). Among them, strands B, C, and D were modified at their 5′ends with disulfide-protected thiol (-SH) groups to enable surface immobilization via Au–S bonds. Each oligonucleotide was dissolved in TE buffer (pH 8.0) to prepare 50 μM stock solutions. To assemble TDNs, 1.0 μL of each oligonucleotide was mixed with 41 μL of TM buffer (20 mM Tris, 50 mM MgCl_2_, pH 8.0) in a centrifuge tube, followed by the addition of 5 μL of freshly prepared TCEP solution (30 mM) to reduce the disulfide-protected thiols. The mixture was heated at 95 °C for 2 min and rapidly cooled to 4 °C within 30 s, yielding a 1.0 μM TDN stock solution. To modify the electrode, 5 μL of the TDN solution was dropped onto the cleaned AuE surface and incubated at 4 °C for 12 h to allow immobilization. After incubation, the electrode was rinsed with double-distilled water to remove unbound or nonspecifically adsorbed DNA. The resulting TDNs/AuE was stored in TM buffer at 4 °C until further use.

### 2.4 Detection of LMP-1

To detect LMP-1, 100 µL of LMP-1 at varying concentrations was added to LMP-1 antibody-coated microplates and incubated at 37 °C for 30 min. After incubation, the plates were washed three times with PBST. Subsequently, 100 µL of detection antibody was added to each well, followed by another incubation at 37 °C for 30 min. The washing step with PBST was repeated three times, and 100 µL of secondary antibody was added. The plates were incubated at 37 °C for another 30 min, and the same washing procedure was performed. Next, 100 µL of streptavidin (SA) solution was added and incubated for 20 min at 37 °C. After washing the microplates, 100 µL of biotinylated SP-HP (1 µM) was added and incubated for 20 min. Subsequently, 100 µL of the SDA reaction mixture was added to each well. The mixture consisted of 0.5×Nicking Endonuclease buffer (25 mM Tris-HNO_3_, pH 7.9, 50 mM NaNO_3_, 5 mM Mg(NO_3_)_2_, 0.5 mM dithiothreitol), 0.4 U µL^-1^ Nt·BstNBI nicking enzyme, 0.05 U µL^-1^ Vent (exo-) DNA polymerase, 1 × ThermoPol buffer (10 mM NaNO_3_, 20 mM NH_4_NO_3_, 20 mM Tris-HNO_3_, pH 8.8, 2 mM Mg(NO_3_)_2_, 0.1% Triton X-100), and 250 µM dNTPs. The reaction was incubated at 53°C for 45 min, followed by heat inactivation at 95°C for 10 min. A 5 µL aliquot of the reaction solution was applied to the TDNs/AuE and incubated for 30 min at room temperature (∼25°C). After rinsing the electrode, 5 µL of Ab-AuPt nanozymes was added and incubated for another 20 min. Finally, the electrode was immersed in TMB substrate solution to measure the electrochemical signal.

### 2.5 Electrochemical measurement

Electrochemical measurements were conducted on a CHI 660E electrochemical workstation using a three-electrode setup, comprising a gold electrode (AuE) as the working electrode, a platinum wire as the counter electrode, and an Ag/AgCl reference electrode. Amperometric detection was carried out in TMB substrate at an initial potential of 150 mV, with the reduction current recorded 200 s after the nanozymes-mediated redox reaction reached equilibrium.

### 2.6 Polyacrylamide gel electrophoresis analysis

A 12% polyacrylamide gel was used for electrophoresis with 1× TBE as the running buffer. DNA samples (10 µL each) were combined with 5× loading buffer and loaded into the assigned wells. The electrophoresis was conducted at a constant voltage of 130 V for 30 min. Gels were stained with GelRed for 15 min, followed by visualization and imaging using a gel documentation system.

## 3 Results and discussion

### 3.1 Assay principle

**FIGURE 1:**
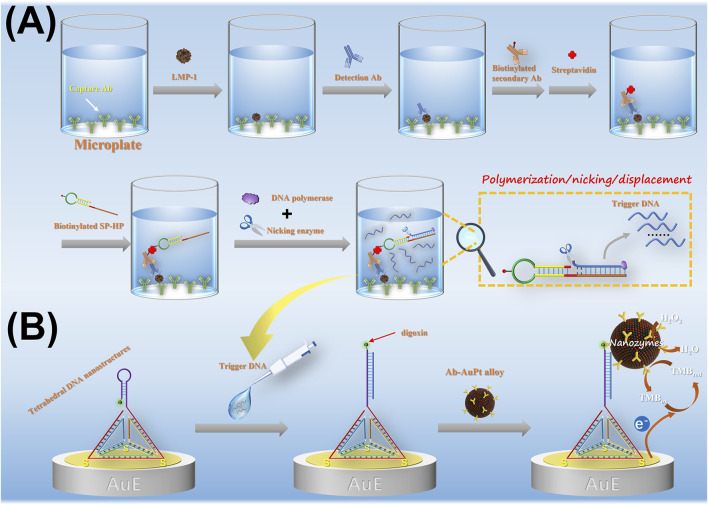
**(A)** Immunorecognition of LMP-1 triggers strand-displacement amplification (SDA). **(B)** Amperometric detection of LMP-1 based on SDA-product–mediated capture of nanozymes by DNA tetrahedra.

**FIGURE 2:**
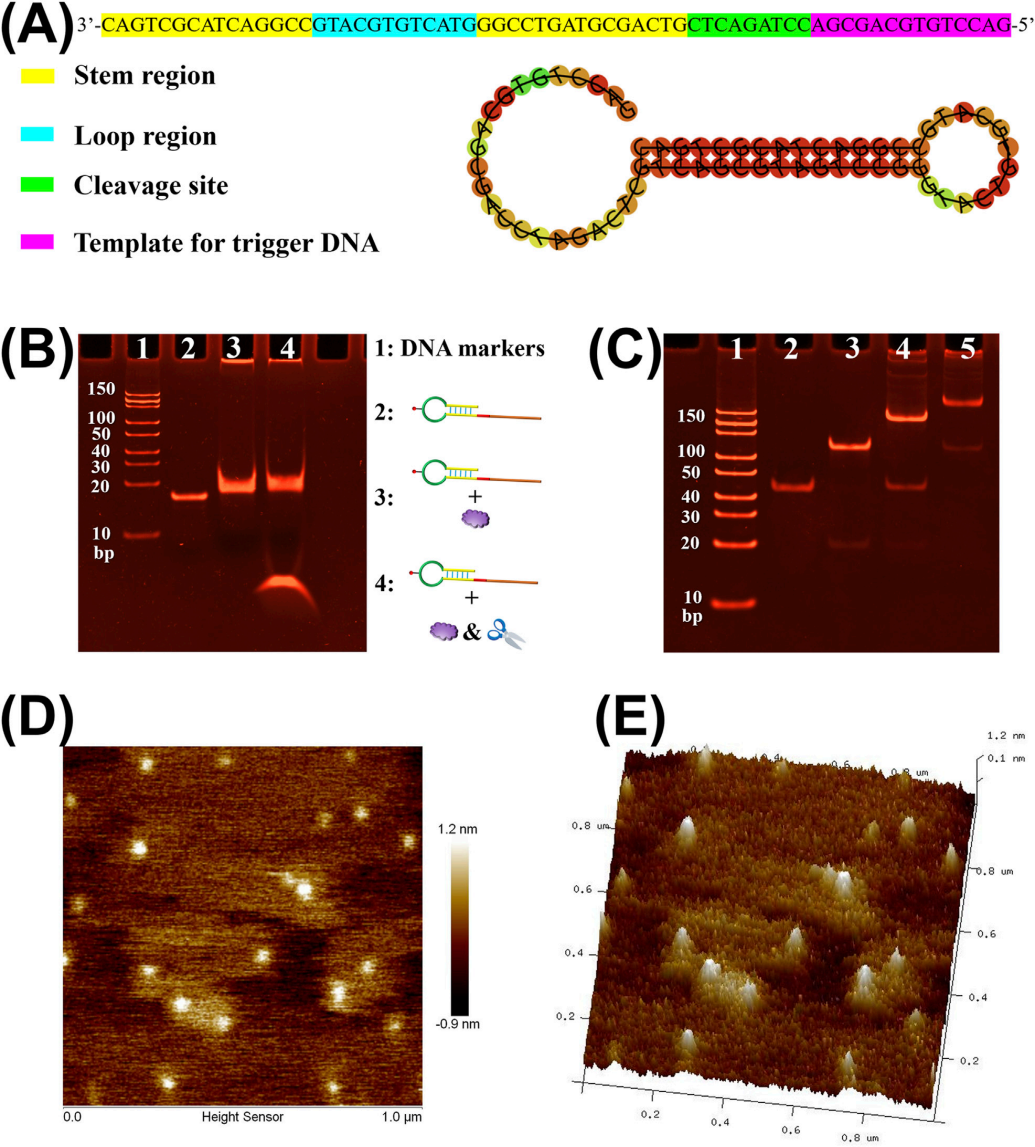
**(A)** The sequence and secondary structure of SP-HP. **(B)** Polyacrylamide gel electrophoresis analysis of the SP-HP-mediated amplification reaction. Lane 1: DNA markers; Lane 2: SP-HP; Lane 3: SP-HP + Vent (exo-) DNA polymerase; Lane 4: SP-HP + Vent (exo-) DNA polymerase + Nt·BstNBI nicking enzyme. **(C)** Polyacrylamide gel electrophoresis analysis of the DNA tetrahedron assembly process. Lane 1: DNA markers; Lane 2: Strand A; Lane 3: Strand A+ Strand B; Lane 4: Strand A+ Strand B+ Strand C; Lane 5: Strand A+ Strand B+ Strand C + Strand D. **(D)** 2D AFM image of DNA tetrahedron nanostructures showing uniform dispersion and particle size. **(E)** 3D AFM image revealing the pyramid-like morphology of the DNA tetrahedra.

The working principle of the amperometric immunoassay method for detecting LMP-1 using SDA-mediated nanozyme capture on DNA tetrahedra-based sensor interface is illustrated in Figure 1. The recognition of LMP-1 is achieved through a sandwich immunoreaction on a microplate. Biotinylated secondary antibodies are introduced into the immunocomplex to incorporate biotin, enabling the capture of SA on the microplate via biotin-SA binding. To enable isothermal signal amplification, we designed a biotinylated SP-HP probe, which can bind to the SA in microplate after immunorecognition. The sequence and secondary structure of SP-HP are shown in Figure 2A. The SP-HP probe consists of four distinct regions: a stem, a loop, the cleavage site for Nt. BstNBI nicking enzyme, and a template region for trigger DNA synthesis. The stem of SP-HP, with a 3′hydroxyl group, directly serves as a primer to activate the polymerization activity of Vent (exo-) DNA polymerase. This polymerase synthesizes a complete double-stranded cleavage site and a trigger DNA fragment. The nicking enzyme Nt. BstNBI introduces nicks at the cleavage site, and Vent (exo-) DNA polymerase, which possesses strand displacement activity, extends from the nick, displacing the downstream trigger DNA fragment. This process generates single-stranded, free trigger DNA fragments. The nicking and polymerization cycle continues, producing a large quantity of trigger DNA fragments. These trigger DNA fragments hybridize with hairpin probes on the TDNs, unfolding the hairpin structure and exposing terminal digoxin groups for binding with the Ab-AuPt nanozymes signal tag. Under the catalytic action of AuPt nanozymes, an electroreduction current of oxidized TMB is generated, providing a quantitative indication of LMP-1 concentration. Notably, the SDA strategy adopted in this study is a linear amplification process, as only a single nicking site is embedded in the SP-HP structure.

### 3.2 Feasibility of the designed SDA reaction

We first employed polyacrylamide gel electrophoresis (PAGE) analysis to validate the designed SP-HP’s ability to mediate strand displacement amplification (SDA) reaction. As depicted in Figure 2B, lane 1 represents the DNA markers, providing molecular weight references for comparison. Lane 2 shows the SP-HP alone, demonstrating its expected molecular size. When SP-HP was incubated with Vent (exo^−^) DNA polymerase and dNTPs, a marked increase in molecular weight was observed (lane 3). This indicates that SP-HP effectively acts as both a primer and a template, initiating the polymerase-mediated amplification process. The interaction between SP-HP and the polymerase highlights its dual functionality, which is crucial for amplification efficiency. To further investigate the SDA process, SP-HP was combined with Vent (exo-) DNA polymerase, Nt. BstNBI, and dNTPs. The results showed not only an increase in SP-HP’s molecular weight but also the emergence of a distinct, smaller, and highly intense band (lane 4). This band corresponds to the trigger DNA fragments generated through SDA. These findings confirm that SP-HP successfully mediates SDA, producing a substantial amount of trigger DNA fragments. This capability underscores the effectiveness of SP-HP in driving signal amplification for downstream electrochemical applications.

PAGE analysis was also employed to characterize the successful synthesis of TDNs. TDNs were assembled from equimolar amounts of four oligonucleotides: strands A, B, C, and D. As shown in Figure 2C, lane 1 represents the DNA markers, which serve as molecular weight references. Lane 2 displays strand A alone, showing its expected molecular size. In lane 3, the mixture of strand A and strand B forms a larger molecular complex, as indicated by the increased molecular weight. Similarly, lane 4 demonstrates the addition of strand C, which further increases the molecular weight of the DNA complex. Finally, when all four strands (A, B, C, and D) were combined, lane 5 reveals a distinct band corresponding to a high molecular weight DNA structure. In addition, atomic force microscope (AFM) imaging was used to directly observe the morphology of the assembled nanostructures. As shown in the 2D AFM image (Figure 2D), the DNA tetrahedra appear as uniformly dispersed, monodisperse nanoparticles. The corresponding 3D topographic image (Figure 2E) reveals a clear pyramid-like shape, which is consistent with the expected geometry of DNA tetrahedra. This result confirms that the TDNs were successfully assembled through the hybridization of the four oligonucleotides.

### 3.3 Characterization of the AuPt alloy nanozymes

**FIGURE 3:**
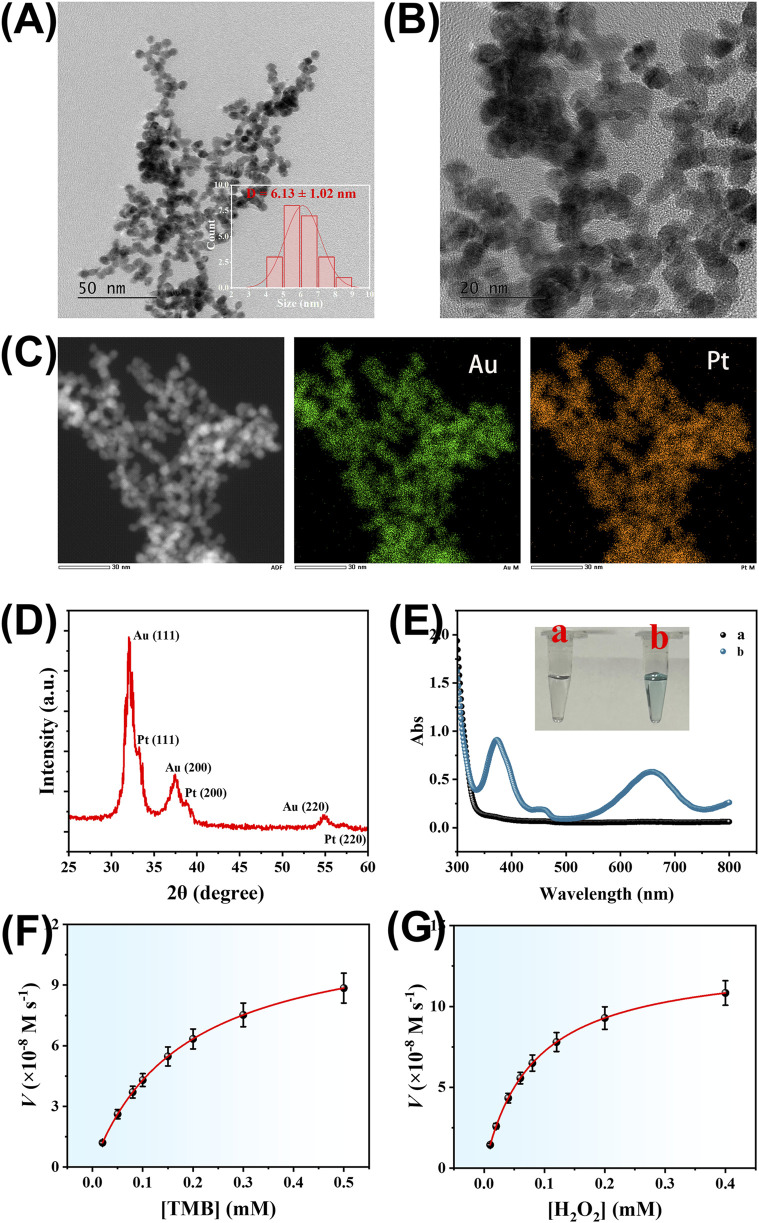
**(A)** Low-magnification TEM images of the AuPt alloy nanoparticles. *Inset*: size distribution histogram. **(B)** high-magnification TEM images of the AuPt alloy nanoparticles. **(C)** High-angle annular dark-field scanning transmission electron microscopy (HAADF-STEM) image and corresponding elemental mapping showing uniform distribution of Au and Pt in the alloy nanoparticles. **(D)** XRD pattern of the AuPt alloy nanoparticles. **(E)** UV–vis absorption spectra of the TMB + H_2_O_2_ system without **(A)** and with **(B)** AuPt alloy nanoparticles. The inset shows the corresponding digital photographs of the reaction solutions. **(F)** Michaelis–Menten plot of the AuPt alloy nanoparticles toward TMB substrate. **(G)** Michaelis–Menten plot of the AuPt alloy nanoparticles toward H_2_O_2_ substrate.

Due to the excellent catalytic activity of Pt and the bioconjugation-friendly properties of Au, AuPt alloy nanozymes were selected as signal amplification elements in this work. The morphology of the synthesized AuPt alloy nanozymes was characterized by transmission electron microscopy (TEM). As shown in Figure 3A, the low-magnification TEM image reveals that the AuPt alloy nanozymes are uniformly dispersed and exhibit a nearly spherical morphology. The inset in Figure 3A presents the particle size distribution histogram obtained by analyzing multiple nanoparticles using ImageJ software. The average diameter was calculated to be 6.13 ± 1.02 nm, indicating a narrow size distribution and good monodispersity. A higher-magnification image (Figure 3B) further confirms their well-defined structure. In addition, a high-angle annular dark-field scanning transmission electron microscopy (HAADF-STEM) image and the corresponding elemental mapping (Figure 3C) confirm the homogeneous distribution of both Au and Pt elements throughout the nanoparticles. These observations collectively verify the successful synthesis of the AuPt alloy nanozymes with a well-defined bimetallic structure. To confirm the crystalline structure and alloy formation of the synthesized AuPt nanozymes, X-ray diffraction (XRD) analysis was performed, and the results are presented in Figure 3D. The XRD pattern reveals distinct diffraction peaks at 32.08°, 33.26°, 37.34°, 38.74°, 54.94°, and 57.14°, which correspond to the (111), (200), and (220) planes of face-centered cubic (fcc) gold and platinum, respectively. The simultaneous presence of characteristic peaks from both Au and Pt confirms the formation of bimetallic AuPt alloy structures rather than physical mixtures.

To evaluate the peroxidase-like catalytic activity of the AuPt alloy nanozymes, UV–visible absorption spectroscopy was employed using TMB and H_2_O_2_ as the substrate system. As shown in Figure 3E, the TMB + H_2_O_2_ system alone exhibited negligible absorbance, indicating that spontaneous oxidation of TMB did not occur under the experimental conditions. However, upon addition of the AuPt alloy nanozymes, two distinct absorbance peaks appeared at 652 nm and 370 nm, which are characteristic of the oxidized form of TMB. The inset photograph in Figure 3E clearly illustrates the visual color change: the TMB + H_2_O_2_ solution remains colorless, while the addition of AuPt alloy nanozymes results in a pronounced blue coloration, further indicating effective catalytic oxidation of TMB. These results demonstrate that the synthesized AuPt alloy nanozymes possess intrinsic peroxidase-like activity. Furthermore, the catalytic affinity of the AuPt alloy nanozymes toward the substrates TMB and H_2_O_2_ was evaluated through steady-state kinetic analysis. As shown in Figures 3F,G, Michaelis–Menten curves were constructed, and the apparent Michaelis–Menten constants (Km) were calculated to be 0.18 mM for TMB and 0.08 mM for H_2_O_2_. These relatively low Km values indicate strong substrate affinities, underscoring the excellent catalytic performance of the AuPt nanozymes and their suitability for application in the developed electrochemical biosensing platform.

To highlight the catalytic superiority of our nanozyme, we systematically compared its substrate affinity with representative reported systems. Fe_3_O_4_ nanoparticles exhibited a Km of 0.098 mM toward TMB/H_2_O_2_ (Zhang et al., 2021), while V_2_O_5_ nanowires showed a Km of 0.12 mM (Niu et al., 2022). Graphene oxide functionalized with carboxyl groups (GO–COOH) presented a higher Km of 0.45 mM (Song et al., 2010), and platinum nanoparticles displayed a Km of 0.60 mM (Fan et al., 2011). MOF-based nanozymes such as Fe-MIL-88NH_2_ and MIL-53(Fe) both demonstrated low Km values of 0.084 and 0.083 mM, respectively (Pargoletti and Gogotsi, 2025). In contrast, the AuPt alloy nanoparticles developed in this work exhibited Km values of 0.18 mM for TMB and 0.08 mM for H_2_O_2_, indicating balanced substrate affinity. These comparative data establish that our nanozyme not only rivals but also surpasses many reported systems, striking an optimal balance between affinity and catalytic efficiency, which is advantageous for sensitive biosensing applications.

### 3.4 Feasibility of the developed amperometric immunoassay method

**FIGURE 4:**
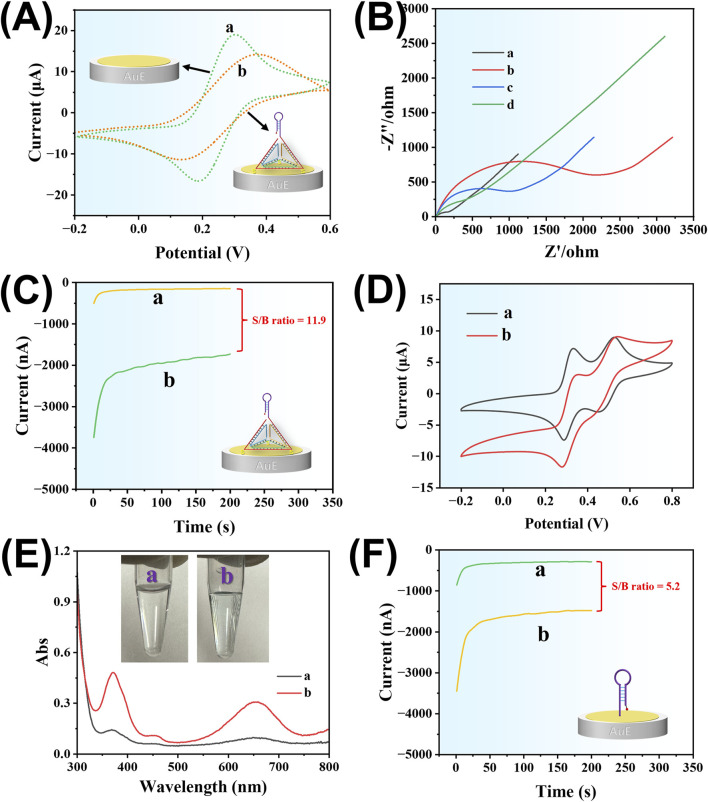
**(A)** Cyclic voltammograms of the AuE before (curve a) and after (curve b) DNA tetrahedron assembly. **(B)** Nyquist plots of EIS recorded at different modification stages of the electrode: (a) bare Au electrode, (b) after TDN immobilization, (c) after incubation with SDA amplification products, and (d) after capture of Ab-AuPt conjugates. **(C)** Amperometric response of TDNs/AuE after incubation with Ab-AuPt in the absence (curve a) and presence (curve b) of amplification products in TMB substrate. **(D)** Cyclic voltammograms of TDNs/AuE after incubation with Ab-AuPt in the absence (curve a) and presence (curve b) of amplification products in TMB substrate. **(E)** UV–vis absorption spectra of TMB substrate after immersion of the sensing electrode: (a) in the absence of LMP-1 and (b) in the presence of LMP-1. The inset shows the corresponding digital photographs of the TMB substrate. **(F)** Amperometric response of HP/AuE after incubation with Ab-AuPt in the absence (curve a) and presence (curve b) of amplification products in TMB substrate.

To investigate the feasibility of the developed method, we utilized cyclic voltammetry (CV) to verify the successful assembly of TDNs on the surface of AuE. Figure 4A illustrates the CV behavior of the electroactive ion pair [Fe(CN)_6_]^3-^/^4-^ at the AuE surface, both before and after TDNs assembly. As shown in curve a, the bare AuE exhibits a pair of well-defined, reversible redox peaks with high peak currents, corresponding to the redox activity of [Fe(CN)_6_]^3-^/^4-^. However, after the assembly of TDNs on the AuE (curve b), both the reversibility and peak currents of the redox peaks are significantly reduced, a phenomenon consistent with previous reports (Dong et al., 2015). This is attributed to the large size of the TDNs, which hinders the diffusion of [Fe(CN)_6_]^3-^/^4-^ from the solution to the electrode surface. Additionally, the negatively charged DNA in the TDNs repels the negatively charged [Fe(CN)_6_]^3-^/^4-^, further impeding its electrochemical activity at the electrode. These results demonstrate the successful assembly of TDNs on the AuE surface.

To monitor the stepwise interfacial construction illustrated in Figure 1B, electrochemical impedance spectroscopy (EIS) was employed. As shown in Figure 4B, the bare AuE exhibited a very low charge transfer resistance (R_et_, curve a), indicative of fast electron transfer at the unmodified surface. After immobilization of tetrahedral DNA nanostructures (TDNs), R_et_ increased significantly (curve b), suggesting the successful formation of a negatively charged DNA layer that hindered electron transfer. Upon incubation with the SDA amplification products, R_et_ decreased by approximately 50% (curve c), which may be attributed to hybridization-induced structural rearrangement, resulting in increased permeability. Further introduction of the Ab-AuPt conjugates led to a substantial reduction in R_et_ (curve d), likely due to the excellent electrical conductivity of the AuPt nanozymes, which facilitated interfacial electron transfer once captured on the electrode. On the other hand, the assembly density of TDNs on the AuE surface was calculated to be 9.7 × 10^9^ molecules cm^-2^ according to chronoamperometry measurements (Steel et al., 1998).

To evaluate the feasibility of the developed sensor for quantitative detection of LMP-1, we utilized the *I*−t curve to monitor the electroreduction current of the TMB substrate catalyzed by AuPt alloy nanozymes. In this experiment, a sandwich immunoreaction was performed with and without the presence of LMP-1, followed by the SDA process. The reaction system in the microplate was then transferred to the surface of the TDNs/AuE electrode. After incubation with Ab-AuPt, the *I*−t curve test was conducted in the TMB substrate solution. As shown in Figure 4C, in the absence of LMP-1, only a small background current was observed (curve a). In contrast, the presence of LMP-1 led to a significant increase in the electroreduction current of TMB (curve b), resulting in a signal-to-background (S/B) ratio of 11.9. This result demonstrates that the developed sensor can effectively detect LMP-1. This phenomenon was further validated by CV in the TMB substrate solution. As shown in Figure 4D, in the absence of LMP-1, two pairs of well-defined redox peaks were observed, which are attributed to the typical two-electron redox process of TMB (curve a). In contrast, in the presence of LMP-1, a pair of asymmetric redox peaks characteristic of an electrocatalytic reaction was observed (curve b). Furthermore, to visually verify the capture of Ab-AuPt onto the electrode surface in response to the presence of LMP-1, a colorimetric assay was performed by immersing the sensing electrode into the TMB substrate solution. As shown in Figure 4E, no color change was observed in the absence of LMP-1, indicating that Ab-AuPt was not immobilized on the electrode. In contrast, a distinct blue coloration appeared in the presence of LMP-1, confirming the successful capture of Ab-AuPt on the electrode surface. The corresponding UV–vis absorption spectra further support this result clearly. These findings further support the specific recognition and signal output capability of the developed method.

To further verify the role of TDNs in enhancing detection performance, the hairpin probe embedded in the TDNs was replaced with a standalone hairpin probe (HP), and the resulting electrode was termed HP/AuE. As depicted in Figure 4F, when HP/AuE was used as the sensor electrode, a low background current was also observed in the absence of LMP-1, and the current increased significantly in its presence. However, the S/B ratio was only 5.2, substantially lower than the 11.9 observed with TDNs/AuE. These results confirm that TDNs effectively improve the sensor’s signal conversion capability and detection performance.

### 3.5 Optimization of experimental conditions

**FIGURE 5:**
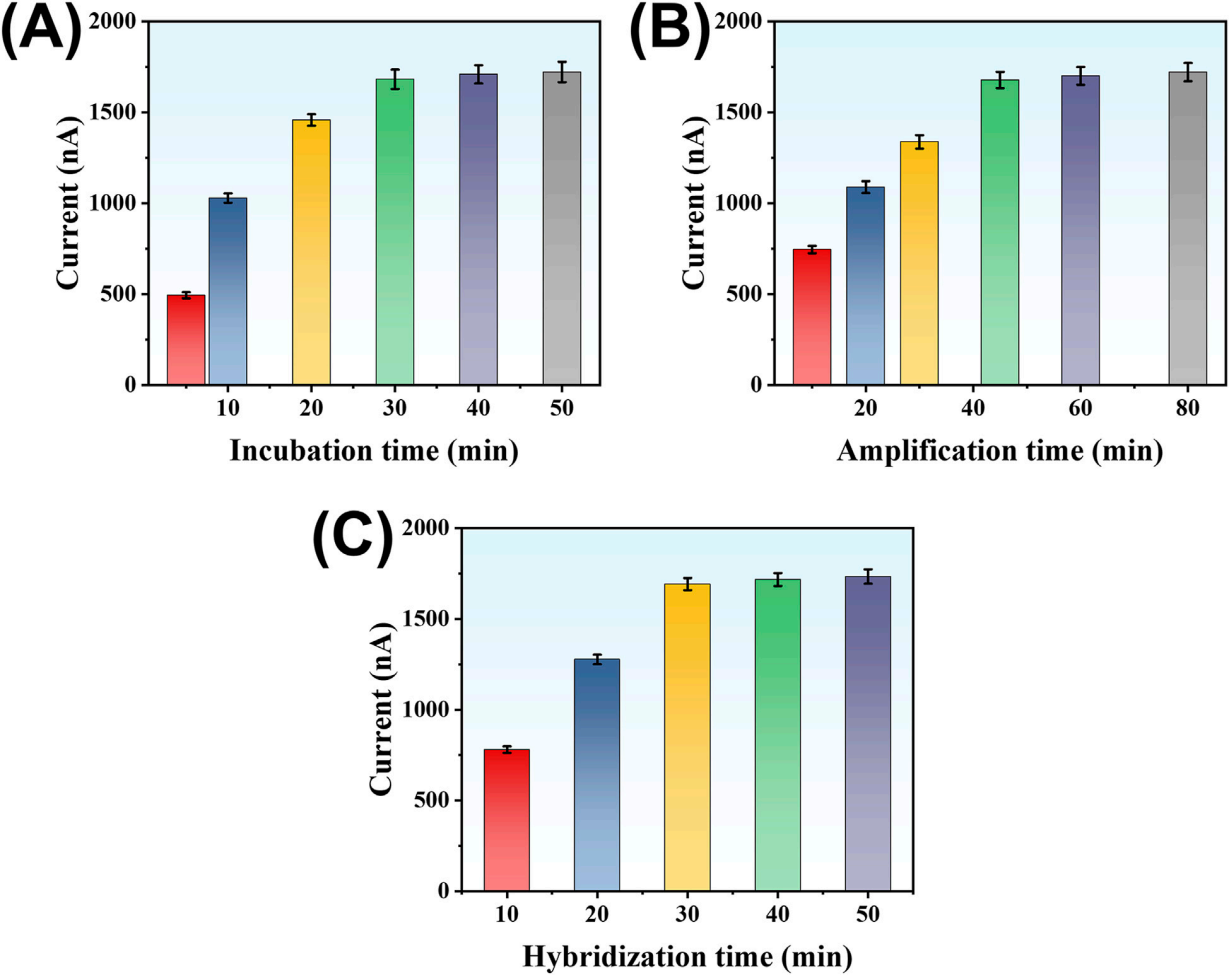
Effect of LMP-1 incubation time **(A)**, reaction time of SP-HP-mediated amplification reaction **(B)**, and hybridization time between Trigger DNA strand and DNA tetrahedron **(C)** on detection signal.

To ensure the highest detection performance, we systematically optimized several experimental parameters, including the incubation time of LMP-1 in the microplate, the duration of the SDA reaction, and the hybridization time between the trigger DNA and the hairpin probe on TDNs. The response signal was quantified by measuring the current output. First, the incubation time of LMP-1 in the microplate was examined. As shown in Figure 5A, the current signal increased as the incubation time was extended, reaching a stable value after 30 min. Extending the incubation time beyond 30 min did not yield further improvements, suggesting that the system had reached saturation. Thus, 30 min was determined to be the optimal incubation time. Next, the SDA reaction time was investigated to maximize signal generation. As illustrated in Figure 5B, the current signal grew steadily as the reaction proceeded, stabilizing after 45 min. This indicates that sufficient trigger DNA fragments were produced within this period. Prolonging the reaction beyond 45 min offered no additional benefits, confirming that the amplification had reached its limit. Therefore, the optimal SDA reaction time was set at 45 min. Lastly, the hybridization time of the trigger DNA with the hairpin probe on TDNs was optimized. Figure 5C shows that the current signal increased with hybridization time, leveling off after 30 min. This stabilization suggests that equilibrium hybridization was achieved, ensuring maximal signal output. Hence, 30 min was selected as the optimal hybridization time.

### 3.6 Analytical performance of the developed amperometric sensor

**FIGURE 6:**
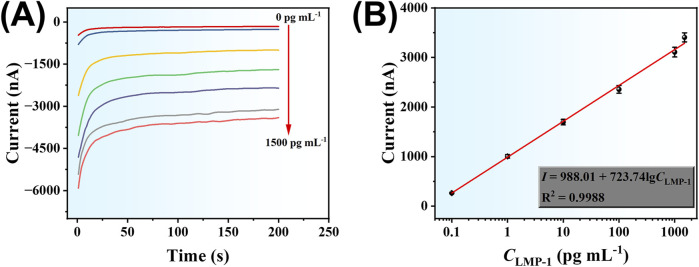
**(A)** Amperometric responses of the developed electrochemical method to different concentrations of LMP-1 (0.1, 1.0, 10, 100, 1,000, 1,500 pg mL^-1^). **(B)** Corresponding calibration curve.

Under optimized experimental conditions, the analytical performance of the developed amperometric immunoassay method for LMP-1 detection was evaluated. As shown in Figure 6A, the absolute current signal increased progressively with rising LMP-1 concentrations in the range of 0.1–1,500 pg mL^-1^, demonstrating the sensor’s responsiveness to varying target concentrations. In addition, Figure 6B illustrates a strong linear relationship between the current response (*I*) and the logarithm of LMP-1 concentration (lgC_LMP-1_). The linear regression equation was determined to be: *I* = 988.01 + 723.74 lgC_LMP-1_, with an excellent correlation coefficient of *R* ^2^ = 0.9988. The detection limit (LOD) was calculated to be 47 fg mL^-1^ based on a signal-to-noise ratio of 3. These results validate the sensor’s broad linear range, high sensitivity, and its capability for accurate quantification of LMP-1, even at ultra-low concentrations.

**FIGURE 7:**
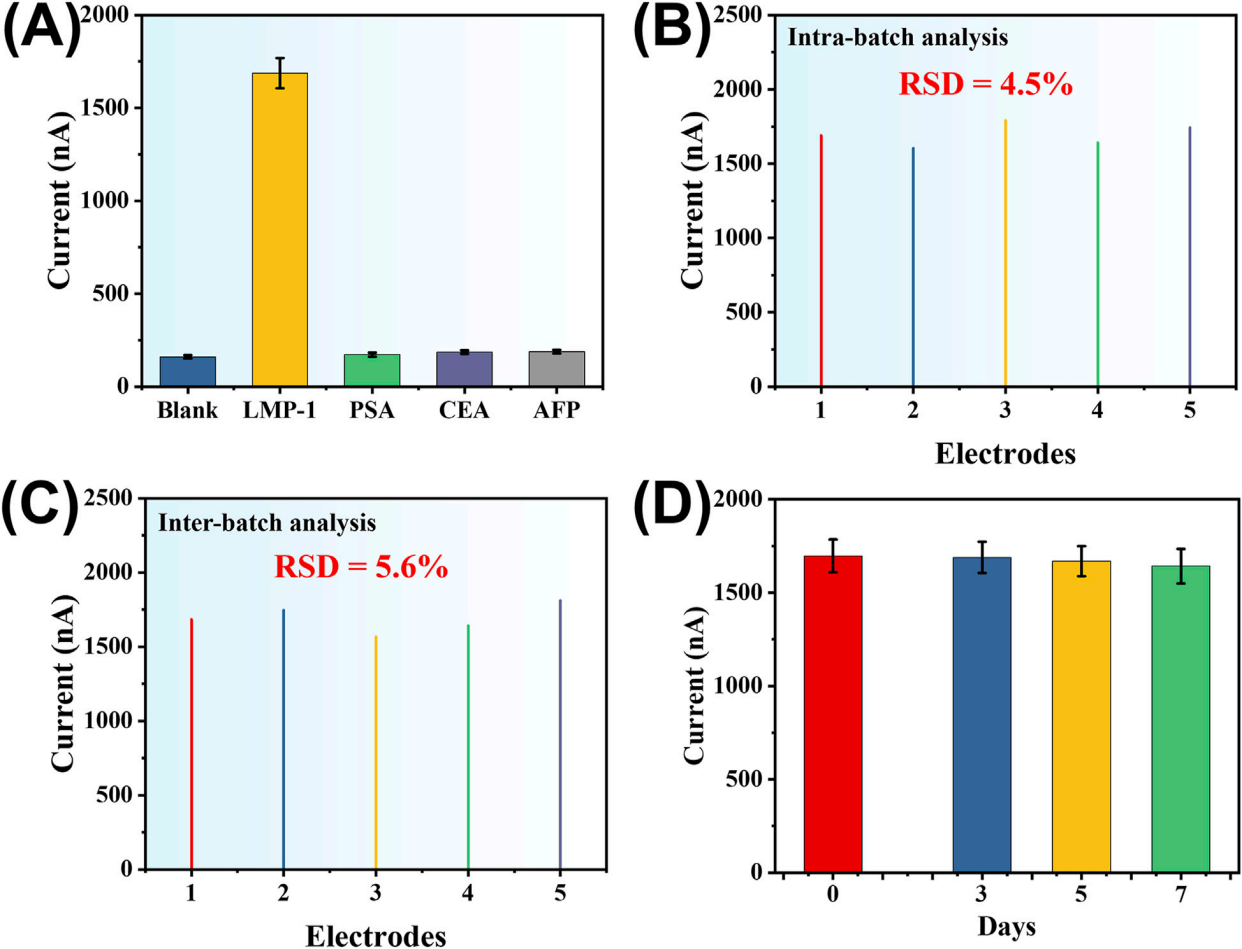
**(A)** Specificity of the developed electrochemical method for LMP-1 compared to PSA, CEA, and AFP (concentration of LMP-1: 10 pg mL^-1^; concentration of interfering proteins: 1 ng mL^-1^). Detection signal of assay systems prepared within the same batch **(B)** and across different batches **(C)**. **(D)** Amperometric responses of TDNs/AuE sensors stored at 4 °C for different durations (0, 3, 5, and 7 days). Measurements were performed using 10 pg mL^-1^ of LMP-1.

The specificity of the developed amperometric sensor for LMP-1 detection was tested against several non-target proteins, including prostate-specific antigen (PSA), carcinoembryonic antigen (CEA), and alpha-fetoprotein (AFP). LMP-1 was used at a concentration of 10 pg mL^-1^, while the interfering proteins were tested at a much higher concentration of 1 ng mL^-1^. As illustrated in Figure 7A, a significant current response was observed exclusively for LMP-1, whereas the signals generated by PSA, CEA, and AFP were negligible. This result highlights the sensor’s excellent selectivity for LMP-1 detection. To evaluate the reproducibility of the developed method, we measured the response signals of assays prepared both within the same batch and from different batches. For the same-batch assays, the relative standard deviation (RSD) of the response signals was 4.5% (Figure 7B). For assays fabricated across different batches, the RSD was 5.6% (Figure 7C). These results demonstrate the sensor’s reliable reproducibility, providing consistent performance across repeated experiments and between fabrication batches. To evaluate the storage stability of the fabricated sensors, TDNs/AuE electrodes were stored at 4 °C and tested over a period of 7 days using 10 pg mL^-1^ of LMP-1. As shown in Figure 7D, the current signal exhibited a gradual and minimal decrease, with the signal retaining 99.5%, 98.4%, and 96.8% of the initial value on days 3, 5, and 7, respectively. These results demonstrate that the sensor retains good stability over a one-week period and is suitable for short-term storage and practical use.

### 3.7 Detection of LMP-1 in spiked fetal bovine serum samples

The practical utility of the developed amperometric immunoassay method was validated by testing its performance in spiked fetal bovine serum (FBS), a complex biological matrix. To create test samples, LMP-1 was spiked into FBS at three concentrations: 1 pg mL^-1^, 100 pg mL^-1^, and 1,000 pg mL^-1^. The prepared samples were then analyzed using the sensor under optimized conditions. As summarized in Table 2, the recoveries for the spiked concentrations ranged from 97.3% to 107.6%, highlighting the method’s accuracy and robustness in detecting LMP-1 within a challenging sample environment. In addition, these spiked samples were also analyzed using an ELISA kit for comparison. The results obtained by the ELISA kit were consistent with those of our method, further confirming the validity and precision of the developed sensor. These results confirm the sensor’s capability for reliable LMP-1 quantification in practical applications, paving the way for its potential use in clinical diagnostics and real-world sample analysis.

**TABLE 2:** Determination of LMP-1 in artificially positive serum samples by using the developed amperometric sensor.

Samples	Spiked (pg mL^-1^)	Found by the sensor (pg mL^-1^)	Recovery (%)	RSD (%, n = 3)	Found by ELISA (pg mL^-1^)
1	1.0	0.97	97.3	5.6	below the LOD
2	100	102.9	102.9	4.9	101.2
3	1,000	1,075.6	107.6	7.2	1,034.6

## 4 Conclusion

In summary, the TDN–SDA–AuPt amperometric immunoassay achieves an LOD of 47 fg mL^-1^ with a 0.1–1,500 pg mL^-1^ linear range for LMP-1. Practically, it combines room-temperature hybridization, isothermal amplification, an enzyme-free AuPt label, and a standard potentiostat; the end-to-end assay time is ∼4 h (electrochemical readout ∼200 s) with routine plate washes—features that lower instrumentation burden and reagent cost versus enzyme/optics-dependent formats. Given the limited number of published LMP-1 sensors, we emphasize the strengths and trade-offs of our platform rather than direct head-to-head comparisons. Limitations include multistep microplate handling, potential TDN instability in high-salt or nuclease-rich matrices, specificity and recovery that still require validation in real clinical serum/plasma beyond spiked FBS, and batch-to-batch variability of AuPt and Ab–AuPt conjugation. Future work will streamline washing/assembly (e.g., magnetic capture or pre-assembled modules), implement batch QC (e.g., TEM/XRD/kinetics), and perform clinical validation to further enhance robustness and translational readiness.

## Data Availability

The original contributions presented in the study are included in the article/supplementary material, further inquiries can be directed to the corresponding author.
